# Assessment of a mouse xenograft model of primary colorectal cancer with special reference to perfluorooctane sulfonate

**DOI:** 10.7717/peerj.5602

**Published:** 2018-11-02

**Authors:** Jeffrey H. Wimsatt, Caitlin Montgomery, Laurel S. Thomas, Charity Savard, Rachel Tallman, Kim Innes, Nezar Jrebi

**Affiliations:** 1 Department of Medicine, West Virginia University, Morgantown, WV, United States of America; 2 Department of Epidemiology, West Virginia University, Morgantown, WV, United States of America; 3 Department of Surgery, West Virginia University, Morgantown, WV, United States of America

**Keywords:** PFOS, Perfluorooctane sulfonate, CRC, PDX, NSG mouse, Patient derived xenograft, NOD SCID Gamma mouse, Treatment, Model selection

## Abstract

Colorectal cancer ranks third among the most commonly diagnosed cancers in the United States. Current therapies have a range of side effects, and the development of a reliable animal model to speed the discovery of safe effective preventative therapies would be of great value. A cross-sectional study in a large Appalachian population recently showed an association between low circulating levels of perfluorooctane sulfonate (PFOS) and a reduced prevalence of colorectal cancer. A study using APC_min_ (C57BL/6J-Apc^Min^/J) mice prone to familial adenomatous polyposis found PFOS was protective when exposure occurred during tumor development. To test the possible benefit of PFOS on spontaneous colorectal cancer, we developed a mouse model utilizing primary patient colorectal cancer implants into NSG (NOD.Cg-*Prkdc^scid^Il2rg^tm1Wjl^*/Sz) mice. Study goals included: (1) to assess potential factors supporting the successful use of colorectal cancer from heterogeneous tumors for PDX studies; and, (2) evaluate PFOS as a therapy in tumor matched pairs of mice randomized to receive PFOS or vehicle. The time in days for mice to grow primary tumors to 5 mm took almost 2 months (mean = 53.3, se = 5.7, range = 17–136). Age of mice at implantation, patient age, gender and race appeared to have no discernable effect on engraftment rates. Engraftment rates for low and high-grade patient tumors were similar. PFOS appeared to reduce tumor size dramatically in one group of tumors, those from the right ascending colon. That is, by 5 weeks of treatment in two mice, PFOS had eliminated their 52.4 mm^3^ and 124.6 mm^3^ masses completely, an effect that was sustained for 10 weeks of treatment; in contrast, their corresponding matched vehicle control mice had tumors that grew to 472.7 mm^3^ and 340.1 mm^3^ in size respectively during the same period. In a third xenograft mouse, the tumor growth was dramatically blunted although not eliminated, and compared favorably to their matched vehicle controls over the same period. These preliminary findings suggested that this mouse model may be advantageous for testing compounds of potential value in the treatment of colorectal cancer, and PFOS may have utility in selected cases.

## Introduction

Colorectal cancer (CRC) ranks third among commonly diagnosed cancers in the United States for both men and women (Cancer, 2017). It is the second leading cause of cancer related deaths in the United States (Cancer, 2017; CDC, 2013). The American Cancer Society estimates that there were 50,260 deaths from colorectal cancer in 2017 (Cancer, 2017). Lifestyle risk factors for colorectal cancer include being overweight, physical inactivity, diets high in red and processed meats, smoking, and heavy alcohol use (Cancer, 2017; CDC, 2013; Haggar & Boushey, 2009). Additional risk factors include age, a history of colorectal polyps, inflammatory bowel disease, and a family history of colorectal cancer (Cancer, 2017; CDC, 2013).

The range of treatments prescribed for CRC is heavily dependent on cancer stage (Cancer, 2017). The three primary treatment modalities are surgery, chemotherapy, and fractionated radiation treatment (CDC, 2013; Haggar & Boushey, 2009). Metastatic disease may require an array of focused and systemic approaches (Sartore-Bianchi et al., 2010). Each of these treatments has various limitations and patients often experience adverse side effects. Drug resistance to chemotherapy is another challenge (Sartore-Bianchi et al., 2010); hence, the development of new agents is highly desirable. For testing novel candidate chemotherapies, preclinical testing in a reliable xenograft (PDX) model can be invaluable if primary tumor heterogeneity is retained (Julien et al., 2012). Here we explore key management considerations using this mouse model while evaluating a candidate chemotherapeutic agent previously associated with a reduced risk of CRC in a human population exposed to background levels (Innes et al., 2014).

Mice used for CRC PDX studies include athymic nude (nu/nu), NOD/SCID (NOD.CB17-Prkdcscid/J or NOD.CB17-Prkdcscid/NcrCrl), BALB/c nude (C.Cg/AnNTac-Foxn1nu NE9), and NSG models (Brown et al., 2016). The immune system of the NSG mouse has been altered to accept a variety of human tumor cells, including solid tumors, without rejection (Shultz et al., 2014). Similarly, NSG mouse use in PDX CRC models, regardless of implantation site, appeared to retain tumor and stromal architecture, cytokine production, and histological morphology (Brown et al., 2016). Recently, NSG mice were used as a chemotherapeutic “avatar” guiding bedside patient care (Garralda et al., 2014). Since the NSG mouse model appears to improve engraftment rates and may better model primary tumors *in situ*, they were selected for study (Puchalapalli et al., 2016; Shultz et al., 2014).

Perfluorooctane sulfonate (PFOS) is a manmade compound that was used in numerous industrial processes (Buck et al., 2011). Prior to 2000, when production was phased out, it was a key ingredient in 3M’s products such as ScotchGuard^®^, used in households to protect upholstery fabrics. It is extremely stable in the environment, and its prolomged environmental persistence has led to its designation as a potential pollutant (Chang et al., 2014; Innes et al., 2014). Both PFOS production facility workers and those who lived near these factories were exposed to varying but sometimes high levels of PFOS (Chang et al., 2014; Innes et al., 2014). Although there have been conflicting results about the effects of fluorinated alkyl compounds that have been studied, PFOS appears to be less toxic in humans than, for example, its sister compound perfluorooctanoate (PFOA), a key component in the synthesis of Teflon^®^ and fire retardants (Chang et al., 2014).

A retrospective cohort study conducted by Innes et al. (2014) investigated the association of prevalent CRC with PFOA, other fluorinated alkyls, and PFOS; the latter was only present at background levels. Perhaps counter to the prevalent view that PFOS is an undesirable environmental contaminant, this study concluded that there was a highly statistically significant inverse dose–response association between low-level (i.e., environmental exposure levels) PFOS serum levels and CRC (Innes et al., 2014). Even so, this retrospective study was not designed to determine if PFOS was causally linked to reduced CRC prevalence, and even if it was protective, the effect was prophylactic or therapeutic in nature (Innes et al., 2014). A recently published paper by our group using the APC_min_ (C57BL/6J-ApcMin/J) mouse model of familial adenomatous polyposis showed that PFOS significantly impeded spontaneous tumor development in male and female mice compared to controls in a dose-responsive fashion (Wimsatt et al., 2016).

In order to test if PFOS might be of benefit in human CRC, preclinical animal safety and efficacy studies are required, utilizing a suitable PDX model (Karim & Huso, 2013). In addition, we assessed the impact of selected methodological attributes on tumor growth characteristics when implanted into NSG mice. Attributes of interest as relates to engraftment efficiency included mouse age, implant origin, patient characteristics, tumor induction times, tumor growth rates, and PFOS exposure outcomes.

## Materials and Methods

### Reagents

PFOS (Sigma #77282; heptadecafluorooctanesulfonic acid potassium salt) 1 mg/ml stock solution was made using Millipore water. PFOS was solubilized with the addition of 0.5% tissue culture grade Tween-20 (Sigma #P2287); Tween-20 in water was used as the vehicle solution.

### Animals

All procedures were approved by the West Virginia University (WVU) Institutional Animal Care and Use Committee (#14-0605) and the WVU Institutional Review Board for human tissue collections. The latter required patient consent. Animals were acquired from the WVU Transgenic Animal Core NSG mouse (origin: NOD.Cg-*Prkdc*^*scid*^* Il2rg* ^*tm*1*Wjl*^/SzJ) breeding colony. Most tumors were obtained overnight by FedEx from NIH sponsored tissue collection consortia, the National Disease Research Interchange (# 1510874473; 6 tumors, Site 1) and Cooperative Human Tissue Network (# 15098320567; 7 tumors, Site 2). Two tumor samples were acquired from the local hospital (WVU IRB #1405312126).

Forty-four NSG mice were used aged 34–103 days (median = 49, range = 69) at the time of implantation. Mice were housed in Techniplast Blueline^®^IVC caging on a 12L: 12D light cycle and fed standard (Envigo, Teklad Global, 2018S) irradiated rodent chow. All food, cages, water, and other items that came in contact with mice were sterile and handled using aseptic technique within a certified biosafety cabinet.

Mice were provided with free access to food and water, and monitored for health twice daily, combined with routine tumor scoring. Drinking water for these mice was supplemented with sulfamethoxazole/trimethoprim (Septra^®^ suspension, total 0.31 mg/ml) on an alternate week basis. This drug was previously shown by us to be soluble with PFOS and Tween-20 in solution. Mice were housed with littermates prior to surgery. After surgery, they were housed together in pairs by tumor, until placed on treatment. Once any mouse developed a tumor diameter of at least 5 mm during weekly scoring, they were placed on study, and single housed to measure PFOS drinking water intake.

### Prior toxicity testing

A preliminary trial was initiated to assess whether PFOS/Tween-20/Sulfa antibiotic toxicity might occur in NSG mice during our proposed 10-week study period (using a cumulative oral target dose of 200 mg/kg of PFOS). Five age-matched NSG mice were fed PFOS while also receiving every other week sulfa antibiotic addition. Five mice received vehicle and sulfa antibiotic. Each week body weights were taken as a reliable indicator of PFOS toxicity, as previously described (Wimsatt et al., 2016).

**Figure 1:**
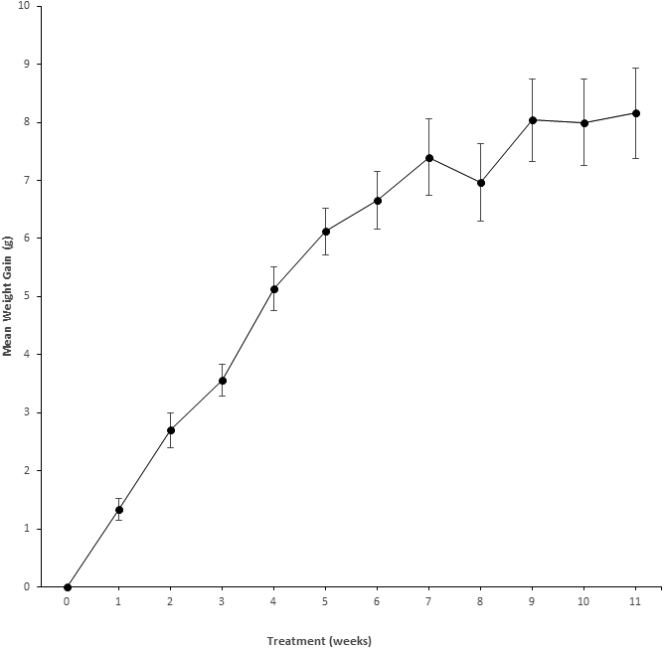
Weight gain in NSG mice exposed to PFOS. Weight gain (mean ± se) over 11 weeks in NSG mice (*n* = 5) receiving PFOS and every other week sulfa drug (6 weeks total) to show there was no significant toxicity. X, Treatment (weeks); Y, Mean weight gain from baseline (g). The abbreviation “se” stands for the standard error.

PFOS was given over 11 weeks (with 6 alternating weeks of sulfa antibiotic). Figure 1 details mouse body weights (mean ± se) from baseline until the end of the exposure period. During routine observation, all appeared to eat well, had expected body weight increases, and remained in excellent body condition for the duration of the treatment period.

### Patient derived xenografts

Human CRC tissues came from patients who had not previously received any therapy; samples were collected at the time of surgical excision. All samples were suspended in cold DMEM solution containing penicillin and streptomycin, packed on ice and transported to our lab by overnight FedEx, or on foot (WVU). Each overnight parcel arrived between 9:30 and 10:15 am the day after collection, and all tumors were immediately implanted into the NSG mice as soon as they became available.

Since successful engraftment rates were unknown prior to the study, four mice from the same litter were allocated to each tumor sample and each mouse received a tumor implant in each flank (eight total implants in four mice per patient), based on an assumed 25% successful implantation rate, and the need for two mice/tumor for study. All surgical procedures took place in a certified biosafety cabinet following ABSL-2 biocontainment procedures. By design, the youngest mouse litter available with 4 mice received tissue implants first. After a brief anesthetic tank induction (5% isoflurane, 3 L O_2_/min), two mice at a time were maintained by mask anesthesia (1.5% isoflurane, 1.5 L/min O_2_) for the procedure duration.

After induction, mice were positioned ventrally recumbent on a heating pad, taped in position, the implantation sites shaved, and skin prepared using a standard aseptic preparation, followed by a final wipe of sterile water and sterile dry swabbing to minimize evaporative cooling.

Extraneous tissues and blood clots were trimmed away from each tumor, before it was sliced into 1 mm wide strips with a sterile scalpel, from which eight 1-mm^3^ pieces were excised for implantation. All tissue pieces were kept wet in cooled DMEM solution until implanted. Using a 14-gauge needle, a subcutaneous pocket was created in each flank extending laterally from a paramedian incision made 1.5 mm from the dorsal midline. Tumors were loaded onto the bevel of the needle and inserted 4 mm into a subcutaneous pocket. Each 2 mm incision was closed with tissue glue (Gluture^®^, Abbott Laboratories). Implantations (4 implants at a time) took no more than 5–7 min after anesthetic induction.

Buprenorphine-SR (2 mg/kg; WildPharm, Windsor, CO, USA) was administered subcutaneously in the neck dorsum during anesthesia. Each mouse was returned to a new cage to recover, and observed closely until fully awake.

### Experimental intervention

Once a tumor reached 5 mm in diameter, 2 mice in each cohort (i.e., matched to a single patient’s tumor) were randomly assigned to receive either PFOS/0.5% Tween-20 or Tween-20 vehicle alone delivered in the drinking water. PFOS was delivered at a target dose of 100 mg/kg over 10 weeks. Water bottle volumes were obtained weekly during bottle changes to track actual PFOS and vehicle water consumption.

If after 5 months no tumor growth occurred, the animal was removed from study. Independent tumor measurements were collected weekly using a digital caliper in triplicate and averaged. Measurements were taken up to 10 weeks after implantation; care was taken to not deform the tumor during measurement. Tumor volume was calculated (volume = 4/3 radius^3^) based on averaging the *x*–*y* dimensions and assuming a spherical shape to the tumors. When tumors developed in both flanks, the first tumor to reach 5 mm was chosen for study. When a single implanted tumor fragment grew as bipartite masses on the same side thus originating from the same implant, the total tumor volume for that side was summed during volume calculations. For analysis purposes, only animals on treatment at least 5 weeks were included.

If an animal scored above 0 using the WVU tumor policy (Table 1) it was removed from study. A matched pair completed study when the second animal scored >0, or when 10 weeks had elapsed. All animals completing the study were humanely euthanized using isoflurane overdose, with death confirmation. Immediately after death, each mouse was necropsied and tissue collected for freezing.

**Table 1:** Humane endpoint tumor policy used in the study to score tumor bearing mice. A score >0 indicated study completion for that mouse.

**Parameter**	**Observation**	**Score**
**General appearance**	**Normal** (e.g., appropriate body condition; healthy appearing fur; pink mucus membranes, bright, alert, responsive)	0
	**Mild Abnormal** (e.g., rough/scruffy fur, slightly decreased activity and grooming, pale mucus membranes)	10
	**Moderately abnormal** (e.g., hunched posture, squinted eyes, reluctant to move, cachectic body condition, white mucus membranes)	20
	**Severely Compromised** (e.g., Minimally to non-responsive, closed eyes)	30
**Body condition score (BCS)**	**Normal to Overweight**—body condition (BCS 3)	0
	**Thin**—obvious dorsal vertebrae (BCS 2)	20
	**Severe Cachexia**—prominent dorsal vertebrae and skeleton (BCS 1)	30
**Tumor appearance**	**Non-ulcerated**—**Not** limiting normal mobility; **Not** limiting ability to eat or breathe	0
	*[One or more listed below]*	20
	**Non-ulcerated wound**—associated with tumor-intact healing or scab present; **Limiting** normal mobility; **Limiting** ability to reach food and /or water	
	*[One or more listed below]*	30
	**Ulcerated or actively bleeding**—**Preventing** mobility, or so cannot eat or drink; **Limiting** ability to breathe, or any combination thereof	
**Respiration**	**Normal** rate and effort for species/strain	0
	**Increased rate and/or effort** for species/strain	30
	**Severe respiratory distress or gasping** (agonal) breathing pattern	60

### Statistical analysis

Demographic patient information, site of origin, and descriptive statistics for patient tumors were tabulated. Summary statistics for mouse parameters, including tumor engraftment rates, time for a tumor diameter to reach at least 5 mm, and mouse age at implantation were determined. Outcomes summarized included weekly water consumption, PFOS consumption and tumor volumes.

For statistical analysis *α* was set to 0.05, and *p* ≤ 0.05 was considered significant. To examine treatment effects through time a repeated-measures ANOVA (treatment = independent variable; weekly growth change = dependent variable) was employed for the initial 5-week treatment period. To explore whether mouse age at implantation related to successful engraftment time, linear regression was applied. Student’s 2-sided *t*-test was used to determine if there was a significant difference in patient ages leading to successful engraftment. Fisher’s exact test was employed to deduce whether there was a difference in proportion of engraftment rates between commercial Sites 1 and 2.

**Figure 2:**
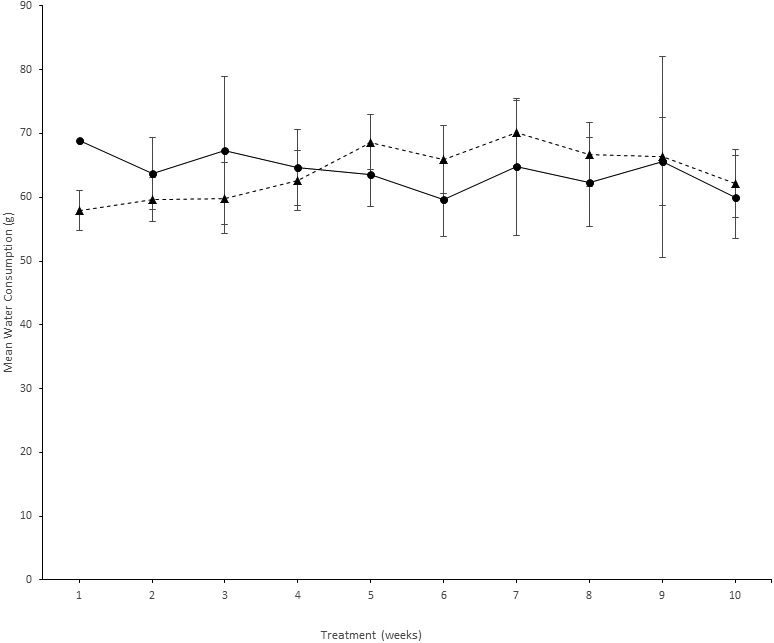
Comparison of PFOS water and vehicle water consumption. Animal water consumption (mean ± se) in the PFOS and vehicle groups over the 10-week treatment period. The *x*-axis depicts treatment (in weeks), and the *y*-axis is the mean water consumption (g). Symbols: *Trianges* depict mice receiving PFOS treatment, and *circular* symbols depict mice receiving vehicle alone. The error bars represent standard errors (se).

## Results

### Toxicity testing

Figure 1 depicts the weight gain profile for PFOS in preliminary NSG mice to indicate that no discernable toxicity was recognized from high dose PFOS with sulfa antibiotic administration. Shown in Fig. 2 is the weekly water consumption (means ± se) for mice on study receiving sulfa antibiotic, and bearing tumors receiving PFOS or vehicle. Overall, the amount of water consumed by both groups was relatively stable. Water consumption by the PFOS group started out lower, but had caught up by 4 weeks of study.

Not all study animals received 10 weeks of treatment if they acquired a humane endpoint criterion >0 first, as described in Table 1. Even so, all animals completed 5 weeks of treatment.

### Patient tumor characteristics

Fifteen patient tumors were received, and of these, eleven exhibited at least 5 mm diameter tumor growth in one or more mice. Six of patient tumors allowed treatment in matched pairs. Descriptive statistics for the patient derived tumors are summarized in Table 2. Mean patient age was 66.6 years (sd = 14.1, min = 42, max = 86), and represented slightly more males (47%) than females (40%). One tumor was a poorly differentiated cecal carcinoma with medullary features, while the 14 others were adenocarcinomas. Of these latter tumors, 67% were low grade, and 33% high grade. Tumors collected were most numerous from the right colon (40%), followed by cecum (20%), left colon (13.3%), sigmoid colon (13.3%), and rectum (13.3%).

**Table 2:** Characteristics of implanted patient donor CRC samples (*n* = 15) are presented. Patient, gender, race, source, tumor origin, tumor grade, if it metastasized in the patient or not, and tumor classification are shown.

			*N*	(%)
**Patient**	Gender	Male	7	(47%)
		Female	6	(40%)
		n/a^a^	2	(13%)
	Race	White	10	(67%)
		Black	1	(7%)
		n/a^a^	4	(27%)
	Source	Site 1	6	(40%)
		Site 2	7	(47%)
		Local hospital	2	(13%)
**Tumor**	Site	Cecum	3	(20%)
		Right colon	6	(40%)
		Left colon	2	(13%)
		Sigmoid colon	2	(13%)
		Rectum	2	(13%)
	Grade	High grade	5	(33%)
		Low grade	10	(67%)
	Metastatic in Patient	Yes	5	(33%)
		No	10	(67%)
	Type	Adenocarcinoma	14	(93%)
		Poorly differentiated carcinoma with medullary features^b^	1	(7%)

**Notes.**

a information unavailable.

b Cecum.

The six tumors tested in matched pairs that grew in mice were from differing locations (ascending right colon 3; cecum 2; sigmoid colon 1). Patient age for successful engraftments (mean = 68.7 y, *n* = 6) were not significantly different (*p* = 0.45) from those that failed to engraft (mean = 62 y, *n* = 9).

### Mouse tumor outcomes

Twenty-eight of the 44 mice (63.6% overall engraftment rate) implanted grew a tumor 5 mm in diameter, however not all these tumors grew in more than 1 mouse (*n* = 2) allowing study; conversely, some tumors grew in >2 mice (*n* = 7). Fourteen mice grew tumors in both flanks, 14 mice grew a tumor on only one side, and 16 mice grew no tumors. Mouse outcomes by source are summarized in Table 3. Mice assigned to implantation surgery averaged 58.8 days of age (se = 8.8, range 32–103). Mouse age at implantation did not appear to affect successful engraftment ((engraft) = 0.098(age) + 59.055; *r*^2^ = 0.03, *p* = 0.65). When all animals growing tumors were considered, the average time for an implanted mouse to grow a tumor to at least 5 mm was 53.3 days (se = 5.7, range: 17–136). The longest pair that successfully engrafted took 126 days. However, most (21/28) tumors engrafted and grew to at least 5 mm by 70 days.

**Table 3:** Summary statistics for implanted mice with at least one tumor over 5 mm (*n* = 11) are presented. Age at implantation, time to reach at least 5 mm (*n* = 28), time between removal of human tumor and mouse implantation (if reported), and engraftment rate by mouse are provided by source.^b^

	**Local**	**Site 1**	**Site 2**	**All**
**Mouse Implantation Age in Days**	35.5 ± 3.5 [32, 39] (*n* = 2)	62.4 ± 15.1 [35, 103] (*n* = 5)	66.0 ± 14.5 [34, 102] (*n* = 4)	58.8 ± 8.8 [32, 103] (*n* = 11)
**Time to Grow to 5 mm Diameter in Days**	71.3 ± 24.6 [17, 136] (*n* = 4)	55.1 ± 6.8 [23, 126] (*n* = 15)	48.9 ± 9.5 [23, 115] (*n* = 9)	53.3 ± 5.7 [17, 136] (*n* = 28)
**Hours from Collection to Implantation**	[2, 4] (*n* = 4)	21.6 ± 2.2 [17.3, 32] (*n* = 15)	Unknown^a^ (*n* = 9)	
**Number Successfully Implanted Mice (%)**	4/8 (50%)	15/20 (75%)	9/16 (56.3%)	28/44 (63.6%)

**Notes.**

a Time to implant information was not available from all sources.

b Four mice received the same tumor in each flank (eight sides/tumor). Mean ± se, [min, max], (*n*, number of mice).

### Role of the source

Of the 15 total tumors, seven had their origin from tissue banking site 2 (47%), six were tissue banking site 1 (40%), and two came from the local hospital (13%). Site 1 trended toward a better engraftment rate than either site 2 or the local hospital. Of occasions where 2 mice successfully engrafted (site 1 = 83%, site 2 = 43%, local = 50%), perhaps due to small samples sizes, site 1 did not have a statistically higher engraftment rate than site 2 (*p* = 0.2983; Fisher’s Exact Test). Likewise, since only two tumors came from a local source, and only 1 developed into a matched pair, comparisons with this source are of limited value.

### PFOS effect on tumor growth

**Figure 3:**
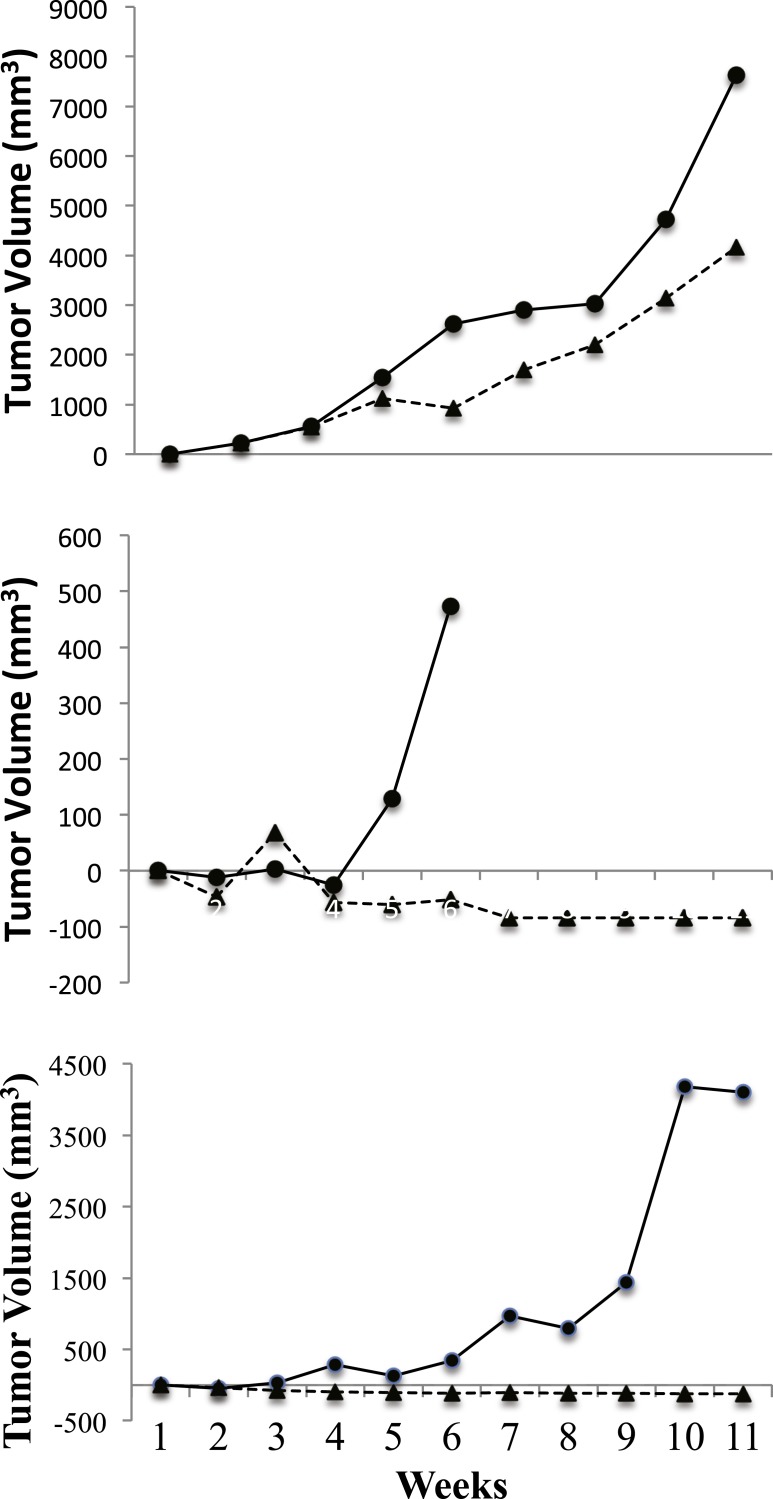
PFOS and vehicle exposed NSG mice that responded. Depicted are**** tumor volumes from baseline through the 10–week treatment period. Represented are three right ascending colon tumors from three different patients. Each frame consists of a matched pair of mice where PFOS (*Trianges*) had a beneficial effect on tumor growth when compared to mice receiving vehicle-only (*circular* symbols). The *X*-axis represents treatment (in weeks), and the *Y*-axis represents tumor growth difference (mm^3^) from baseline tumor growth. The length of time mice remained on study was determined using the tumor policy in Table 1.

**Figure 4:**
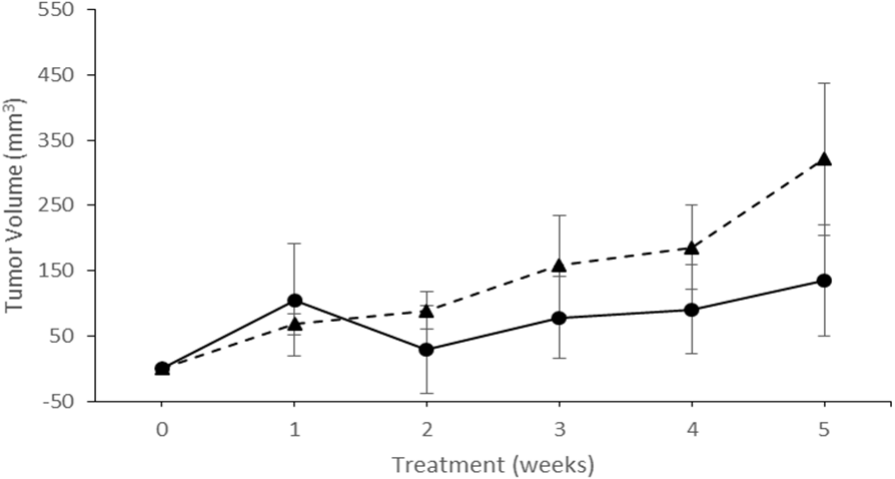
PFOS and vehicle fed NSG mice showing tumor growth in the non-responder group. Differences in calculated tumor volumes (mean ± se) from baseline in study animals over 5 weeks of treatment. All patient tumor implanted into NSG mice were from colon locations other than the ascending right colon (Sigmoid colon, *n* = 1; Cecum, *n* = 2). The *X*-axis represents treatment (in weeks), and the *Y*-axis depicts tumor growth in mm^3^ from baseline tumor growth. Symbols: *Trianges* represent animals receiving PFOS, and *circular* symbols represent mice that received vehicle alone. Animals were removed from study according to the criteria listed in Table 1.

When all the matched pairs were considered together, there was no significant growth difference effect (*p* = 0.45). Tumor volumes as described by donor tumor location are displayed in Figs. 3 and 4. The graphs in Fig. 3 depict each mouse pair from the right ascending colon (*n* = 3 pairs). All tumors from the right ascending colon showed a marked PFOS response. The most dramatic responses were in two cases when PFOS caused the tumor to become grossly undetectable, while the same tumor in its vehicle treated mate continued to grow. In the third graph (top frame)*,* PFOS noticeably blunted tumor growth in response to PFOS as compared to the vehicle control mouse. To show that there was no emergent tumor resistance developing in the PFOS responders through time, tumor volumes for the three pairs of mice from the right ascending colon were followed for the full 10 weeks of treatment.

In Fig. 4, the calculated tumor volume (mean ± se) for matched pairs from tumor origins other than the right ascending colon (sigmoid colon, *n* = 1; cecum, *n* = 2) are shown, and clearly exhibited no PFOS treatment response. Instead, tumor volumes for the PFOS group trended slightly larger than for the vehicle group over 5 weeks of treatment. At necropsy, neither liver metastasis nor liver enlargement were detected from any mouse.

## Discussion

Although sample sizes are small, 50% of tumors tested appeared PFOS responsive, and all were from the right ascending colon. Even so, rodent models suggest PFOS toxicity might be a concern. However, nonhuman primate PFOS kinetics studies suggest that they may better model PFOS disposition in humans, and both humans and model primates may handle the compound quite differently from rodents where most of the toxicity work has been done (Andersen et al., 2006; Chang et al., 2012; Tan, Clewell 3rd & Andersen, 2008). This is particularly true in regard to renal PFOS disposition and P450 enzyme inhibition (Andersen et al., 2006). Accordingly, liver binding of PFOS in rats was demonstrated to be much higher than in nonhuman primates (Tan, Clewell 3rd & Andersen, 2008). Hence, it is possible that PFOS may be more toxic in rodents than in humans due to the generation of toxic metabolites in the former. Likewise, human occupational exposure studies may better reflect the true risk of PFOS exposure in a potential clinical setting (Chang et al., 2014; Karim & Huso, 2013). These latter studies seem to complement what Innes et al. (2014) found, namely that low environmental background levels were associated with reduced CRC in the Ohio Valley in the absence of obvious adverse effects.

Negative human effects of PFOS previously posited include cancers of the prostate, kidney, testis, and thyroid (Chang et al., 2014); even so, the association with cancer was weak at best (Chang et al., 2014). Moreover, these effects were typically from long-term exposures at doses up to 40 times higher than the levels Innes et al. (2014) associated with reduced CRC risk. In a recent reexamination of earlier studies, Arrieta-Cortes and associates (Arrieta-Cortes et al., 2017), suggested that PFOS should not be categorized as a carcinogen. For example, PFOS exposed factory workers with markedly higher circulating levels exhibited surprisingly few adverse outcomes (Grice et al., 2007). Even so, lasting effects in humans have not been well established for low level PFOS exposures (Butenhoff, Olsen & Pfahles-Hutchens, 2006). In any case, many standard chemotherapy agents are toxic and can have serious side effects, while still being of considerable therapeutic value.

PDX models of CRC have been designed to look at an array of questions including for biomarker discovery (Bertotti et al., 2011; Isella et al., 2015; Julien et al., 2012), drug discovery (Dangles-Marie et al., 2007; Puig et al., 2013), model validation (Monsma et al., 2012), and to investigate fundamental questions in tumor biology (Brown et al., 2016; Isella et al., 2015). However, these studies have not typically examined potential factors involved with successful primary heterogeneous PDX implantation from varied commercial sources in the NSG mouse model.

### Model considerations

Here, in all three cases where right ascending colon origin CRC engrafted successfully, there was a marked reduction in tumor growth in response to PFOS exposure. Grossly, complete disappearance occurred in two of these cases. It is interesting that tumors with an origin in the upper right colon that responded also are more likely to have a familial basis (Vasen et al., 2008). Right-sided tumors are often more aggressive and common in women (Ahlquist et al., 2008). Whether a genetic basis to the response to PFOS is relevant, these findings complement our earlier findings in another established genetic (Familial adenomatous polyposis) model of CRC (Wimsatt et al., 2016). Further studies are required to confirm these findings. Of interest is the importance of colorectal stem cancer cells and the potential for chemopreventive therapies for intervention, particularly in hereditary forms of CRC (Kim, 2014).

It is important to note that we do not know the prior PFOS exposure profiles of patients donating tumors to this study. PFOS was removed from production in 2000 (EPA, 2017) in the US, and the EPA has recorded diminishing serum PFOS levels over time. Even so, one could hypothesize that differential responses of our patient tumors may relate to prior environmental exposures, and similarly, if PFOS has anti-tumor properties, that a segment of the population may already be benefitting. In either case, prior exposure could bias against detecting a PFOS anti-cancer benefit.

Fortuitously collected primary patient CRC explants, particularly from commercial sources, could introduce some unique circumstances that might influence their experimental value, including suboptimal handling, potentially lower engraftment rates, and increased costs related to long latencies for tumors to engraft. In the present series, over half of the tumors did not engraft in at least two mice allowing study, even though redundant mice and sites were implanted. These results also suggest that 70 days may be a reasonable period to wait to determine if engraftment will be successful, to conserve money and resources.

The population of tumors engrafted here were diverse with regard to histopathological classification, demographic factors, likely genetic background, and colon location. However, the inherent diversity represented is useful in testing for novel therapies. Although the numbers are small, variation in tumor handling did not seem to have a discernable effect on engraftment success, and the time delay to implantation per se was not predictive of engraftment failure in those cases where time to implantation from surgery was available. It is also worth noting that there is no way to determine if failure to engraft influenced PFOS outcomes by removing certain tumor biotypes from study.

A previous study in nude mice looked at optimization of “take rates” using cryogenically frozen colorectal carcinomas with the aid of Matrigel^®^. In their study, Matrigel^®^ significantly improved their overall take rate to 70% (Gock et al., 2016). In another study using nude mice, xenografts successfully established 62.2% of the time. Successful engraftment was associated with advanced stage (*p* < 0.001) and moderate/poor differentiation of the implanted tumors (*p* = 0.029) (Oh et al., 2015). NOD-SCID and nude mice were employed in a comparison of fresh and cryopreserved tumor implantation rates. Overall there was no significant difference (fresh, 74%; cryopreservation, 71%) (Linnebacher et al., 2010). However, this same study revealed that nude mice poorly retained primary tumor characteristics; hence, a study in NSG mice was preferred here (Brown et al., 2016). Orthotopic implantation is often assumed to better model human cancer, since the mouse is implanted with a human tumor of the same origin in the same location; however, colon inoculation in mice is more invasive, prone to obstruction, labor intensive, and potentially difficult to monitor longitudinally. As a model system, it is difficult to assess if the mouse intestine provides the same microenvironment (microbiome and endogenous milieu) provided in humans.

Subcutaneous PDX implantation has been used elsewhere (Karim & Huso, 2013), and had the convenience of direct longitudinal visualization to determine outcomes. In vivo imaging with fluorescent or chemiluminescent probes might be used longitudinally if the signal to noise ratio is sufficient; however, selective markers may not perform uniformly across a heterogeneous population of patient tumors, making projected comparisons more difficult. The improved resolution of micro- MRI or CT methods can mitigate some of the limitations IVIS imaging presents in PDX models (Karim & Huso, 2013).

### PFOS effects

This preliminary study tested PFOS for human anti-CRC tumor activity. The mechanism driving a beneficial PFOS effect is unknown. Possible candidates could be the effect this compound has on the peroxisome (PPAR) system, or via Nκβ mediation (DeWitt et al., 2009; Takacs & Abbott, 2007). PFOS appears to be a potent PPAR ligand with a demonstrated anti-inflammatory effect both *in vitro* and in animal studies (DeWitt et al., 2009; Rayburn, Ezell & Zhang, 2009; Takacs & Abbott, 2007). Other hypotheses for PFOS action include anti-inflammatory effects on prostanoid pathways (e.g., Prostaglandin H synthase or PLA2) (Deng et al., 2015), or through the Wnt canonical cascade (Brembeck et al., 2011). Finally, recent cell work suggests PFOS may facilitate apoptosis via a mitochondrial dependent mechanism, conceivably speeding errant cell turnover (Wang et al., 2013).

In the present study, it is possible that higher doses could have provided a greater effect; nor did the study determine lower dose limits. Preliminary dosing of NSG mice suggests they were tolerant of PFOS exposures encountered here. To mimic the likely route of exposure in humans (Innes et al., 2014), PFOS was provided ad lib in the drinking water; thus the amount of PFOS consumed had to be projected and varied somewhat among animals. There are inevitable limitations in the methodology employed here in this preliminary study. The spherical assumption for calculating tumor volumes potentially leads to exaggerated tumor volume differences, especially for small tumors, although, this bias was systematically applied across all the data. During measurements, care to avoid tumor compression, and to account for any fluid accumulation around a tumor if present is required. When calculating tumor volumes from averaged surface radius measurements, small measurement errors enlarge when estimating volumes from linear dimensions.

Using preserved replicate primary frozen tumors from successful implantations for *in vivo* and *in vitro* work on treatment responsive control and treatment non-responsive tumors would greatly extend the utility of the present model system. By doing so, a more consistent and cost-effective picture might emerge regarding tumor behavior than was demonstrated working with fortuitous CRC samples alone. The genetic and proteomic characterization of tumors responsive and unresponsive to PFOS should eventually help to identify working hypotheses to address the differential efficacy of PFOS on a subset of tumors, and ideally would lead to new therapeutic venues in the future. Combined measurements of existing PFOS serum levels in patients at the time of tissue collection might further clarify relationships and help explain increased or decreased PFOS activity in CRC samples when tested.

Metabolism of PFOS is considerably more rapid in mice than in humans. Previous studies indicate that the half-life of PFOS is around 40 days in mice (Chang et al., 2012), while the half-life in humans is 4–5 (+) years (Olsen et al., 2007). Hence, PFOS circulating levels may not have fully stabilized in the animals under study. Consistent with epidemiological findings, it may be that prolonged low dose human exposures over years contributed to a prophylactic CRC benefit revealed in that study; a scenario not tested here (Innes et al., 2014). Even so, there did appear to be a marked PFOS therapeutic response in a subset of PDX study animals. These findings combined with the human epidemiological and APCmin study outcomes previously reported, suggest that PFOS may offer a new treatment modality, not only in regards to the compound itself, but also as a basic platform for novel CRC drug discovery.

##  Supplemental Information

10.7717/peerj.5602/supp-1 Data S1 Raw data

## Additional Information and Declarations

### Data Availability

The following information was supplied regarding data availability: The raw data is available as Data S1.
